# Iron Status and Risk of Stroke: A Mendelian Randomization Study

**DOI:** 10.1161/STROKEAHA.118.022701

**Published:** 2018-10-25

**Authors:** Dipender Gill, Grace Monori, Ioanna Tzoulaki, Abbas Dehghan

**Affiliations:** 1 From the Department of Biostatistics and Epidemiology (D.G., G.M., I.T., A.D.), Imperial College London, United Kingdom; 2 MRC-PHE Centre for Environment (I.T., A.D.), Imperial College London, United Kingdom; 3 School of Public Health, and Department of Stroke Medicine (D.G.), Imperial College London, United Kingdom; 4 Department of Hygiene and Epidemiology, University of Ioannina Medical School, Greece (I.T.).

**Keywords:** diet, ferritin, iron, stroke, transferrin

## Abstract

**Background and Purpose—:**

Both iron deficiency and excess have been associated with stroke risk in observational studies. However, such associations may be attributable to confounding from environmental factors. This study uses the Mendelian randomization technique to overcome these limitations by investigating the association between genetic variants related to iron status and stroke risk.

**Methods—:**

A study of 48 972 subjects performed by the Genetics of Iron Status consortium identified genetic variants with concordant relations to 4 biomarkers of iron status (serum iron, transferrin saturation, ferritin, and transferrin) that supported their use as instruments for overall iron status. Genetic estimates from the MEGASTROKE consortium were used to investigate the association between the same genetic variants and stroke risk. The 2-sample ratio method Mendelian randomization approach was used for the main analysis, with the MR-Egger and weighted median techniques used in sensitivity analyses.

**Results—:**

The main results, reported as odds ratio (OR) of stroke per SD unit increase in genetically determined iron status biomarker, showed a detrimental effect of increased iron status on stroke risk (serum iron OR, 1.07; 95% CI, 1.01–1.14; [log-transformed] ferritin OR, 1.18; 95% CI, 1.02–1.36; and transferrin saturation OR, 1.06; 95% CI, 1.01–1.11). A higher transferrin, indicative of lower iron status, was also associated with decreased stroke risk (OR, 0.92; 95% CI, 0.86–0.99). Examining ischemic stroke subtypes, we found the detrimental effect of iron status to be driven by cardioembolic stroke. These results were supported in statistical sensitivity analyses more robust to the inclusion of pleiotropic variants.

**Conclusions—:**

This study provides Mendelian randomization evidence that higher iron status is associated with increased stroke risk and, in particular, cardioembolic stroke. Further work is required to investigate the underlying mechanism and whether this can be targeted in preventative strategies.

## Abstract 2

Supplemental Digital Content is available in the text.

Stroke is the second leading cause of death worldwide, with an age-standardized mortality rate of 86.5 per 100 000 population per year.^1^ Iron has an essential role in many physiological processes, including erythropoiesis, immunity, and oxidative metabolism.^2^ The evidence surrounding the association between iron status and stroke risk is conflicting. Several observational studies have found a link between low iron levels and an increased risk of stroke,^3–5^ whereas other studies support a link between higher iron status and an increased risk of stroke.^5–7^ Furthermore, some studies have identified no relationship between iron status and stroke risk.^8–10^

Genetic variants that are related to an exposure of interest can be used as instruments to investigate the effect of varying that exposure.^11^ The Mendelian randomization (MR) technique exploits this through the random allocation of genetic variants.^11^ Where observational studies investigate the association between an exposure and outcome, MR investigates a link between a genetic variant, such as a single-nucleotide polymorphism (SNP) related to the exposure, and the outcome.^11^ By studying the genetic variants rather than the exposure directly, MR overcomes the potential impact of confounding factors on the exposure and outcome.^11^ MR, therefore, offers advantages in its ability to draw conclusions about the causal relationship between exposure and outcome.

The genetic variants investigated in an MR study must be associated with the phenotype of interest, which is iron status in our present study, a clinically relevant and modifiable trait. Biomarkers of iron status offer a quantifiable phenotype for iron status and include serum iron, ferritin, transferrin, and transferrin saturation.^12^ The genetic instruments used for systemic iron status should have a concordant relation to these biomarkers, by consistently increasing serum iron, ferritin, and transferrin saturation and decreasing transferrin levels to represent an increase in systemic iron levels.^12^ In this way, we use MR to investigate the causal effect of iron status on risk of stroke, building on the previous evidence from observational studies in this area and overcoming the limitations of confounding from environmental factors.

## Methods

The specific data used in this work can be requested from the corresponding author. Only summary data from published studies were used. Appropriate patient consent and ethical approval were obtained in the original studies.

### SNP-Iron Status Biomarker Association Estimates

A meta-analysis of genome-wide studies performed by the Genetics of Iron Status Consortium was used to obtain association estimates between SNPs and biomarkers of iron status.^13^ Data from 11 discovery cohorts and 8 replication cohorts were used in the meta-analysis, which combined data from 48 972 European subjects. Adjustments were made for age and principal component scores, with analysis performed separately for males and females before combining estimates.

Increased serum iron, ferritin, and transferrin saturation and decreased transferrin are associated with increased systemic iron status.^12^ Therefore, these biomarkers were used as surrogate measures of systemic iron status, which is the exposure of interest in this study. To be included in our main analysis, SNPs were selected by their genome-wide significant association with increased serum iron, ferritin, and transferrin saturation and decreased transferrin levels, to represent increased systemic iron status.^14^ The aforementioned meta-analysis by the Genetics of Iron Status consortium found that 11 loci related to these biomarkers of iron status at genome-wide significance (*P*<5×10^-8^; Table I in the online-only Data Supplement).^13^ Of these, 3 loci were associated with concordant changes in all 4 biomarkers at genome-wide significance, indicating a consistent association with systemic iron status (Table): rs1800562 and rs1799945 in the hemochromatosis (*HFE*) gene and rs855791 in the transmembrane protease, serine 6 (*TMPRSS6*) gene.^13^ Therefore, these SNPs were included in the main MR analysis as instruments to represent systemic iron status. There was low linkage disequilibrium between the rs1800562 and rs1799945 SNPs in the *HFE* gene (linkage disequilibrium, *r*^2^<0.01),^13^ consistent with the loci being effectively randomly associated with respect to each other.^15^

**Table.**
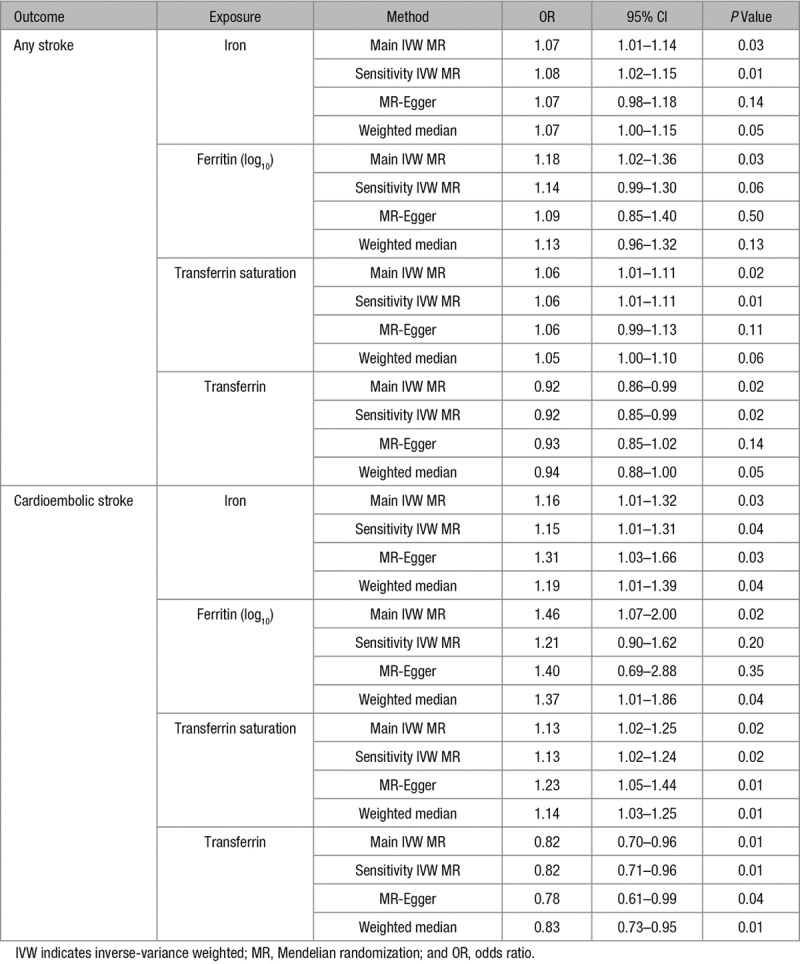
MR Statistical Sensitivity Analyses

The first-stage regression, or F, statistic was used to assess the strength of the instruments and was calculated using the following equation: F=(*R*^2^/*k*)/([1−*R*^2^]/[*n*−*k*−1]), where *R*^*2*^ is the proportion of the iron status variability accounted for by the SNP, *k* is the number of instruments used in the model and *n* is the sample size.^15^

### SNP-Stroke Association Estimates

A meta-analysis of genome-wide association studies was used to derive the association estimates between the SNPs and stroke risk.^16^ Data from the 29 studies included in the MEGASTROKE consortium were used in the meta-analysis, including 67 162 stroke cases, of which there were 60 341 cases of ischemic stroke and 454 450 controls. Subjects were of European, East Asian, South Asian, Mixed Asian, African, and Latin American ancestry. When considering only European participants, there were 40 585 cases of any stroke and 406 111 controls. Furthermore, for ischemic stroke subtypes (of any ethnicity), there were 9006 cases of cardioembolic stroke, 6688 cases of large artery stroke, and 11 710 cases of small vessel stroke.^16^ All of the included studies accounted for age and sex as covariates. Stroke cases were defined using the World Health Organization criteria of sudden development of signs of neurological deficit lasting >24 hours with a vascular cause, with subtypes categorized using the Trial of ORG 10172 in Acute Stroke Treatment criteria.^16,17^

### MR Estimates

Fixed-effect inverse-variance weighted (IVW) meta-analysis of MR estimates derived using the ratio method was used to generate the main MR estimates for the effect of each measure of iron status on risk of any stroke, any ischemic stroke, any stroke limited to European populations, and ischemic stroke subtypes.^15,18^ Standard errors were calculated using the Delta method.^18^ A threshold of *P*<0.05 was used to determine statistical significance. No correction was made for multiple testing because the 4 iron status biomarkers are all used in investigation of the same exposure: overall iron status. Furthermore, multiple testing corrections for sensitivity analyses in the European population and subgroup analyses were not made, as these were only performed to validate the main findings.

### Investigation of Pleiotropy

MR may be confounded by pleiotropic pathways, where genetic variants affect the outcome independently of the exposure of interest.^11^ To investigate this, the PhenoScanner database, which records SNP-phenotype associations (www.phenoscanner.medschl.cam.ac.uk/phenoscanner) was used to check whether the 3 SNPs used in the main analysis had any secondary phenotypes associated with them at genome-wide significance (*P*<5×10^-8^).^19^

To test that our findings were robust to the possible inclusion of pleiotropic SNPs, we relaxed the selection criteria for instruments to include more SNPs and allow statistical sensitivity analyses. Specifically, we included all SNPs in the Genetics of Iron Status Consortium’s GWAS meta-analysis (Genome-Wide Association Study) that were associated with any 1 iron status biomarker at genome-wide significance that also had associations with the other iron status biomarkers in a direction concordant with an effect on overall iron status, even if these did not reach genome-wide significance.^12,13^ This identified a further 3 SNPs for use as instruments: rs9990333 in the transferrin receptor (*TFRC*) gene, rs7385804 in the transferrin receptor 2 (*TFR2*) gene, and rs411988 in the testis expressed 14, intercellular bridge forming factor (*TEX14*) gene (Table I in the online-only Data Supplement). Using this set of 6 instrument SNPs, we repeated the main IVW MR analysis for risk of any stroke and risk of any ischemic stroke subtype that showed statistically significant associations in the main analysis. In addition, we also used these 6 SNPs to perform the MR-Egger and weighted median MR statistical sensitivity analyses, which are more robust to the inclusion of pleiotropic instruments.^20,21^ Given the lower statistical power of these approaches and that they included instruments selected using less robust criteria compared with the main analysis, they were only used to confirm a consistent effect estimate to that seen in the main IVW MR, rather than ascertain statistical significance themselves through any given *P* value threshold.

Data analysis was performed using the statistical program R (version 3.4.3; The R Foundation for Statistical Computing). The data used in this study is publicly available, and the relevant ethical approval was obtained in the original studies.^13,16^

## Results

The SNP-iron biomarker associations are shown in Table I in the online-only Data Supplement. Genetic association estimates for the rs1800562 SNP were not available for small vessel stroke, and neither was a suitable proxy (with linkage disequilibrium *r*^2^>0.3) available. The F statistics for the 3 main SNPs were between 47 and 2127 across all 4 biomarkers of iron status, making significant bias from use of weak instruments unlikely.^15^

**Figure 1.**
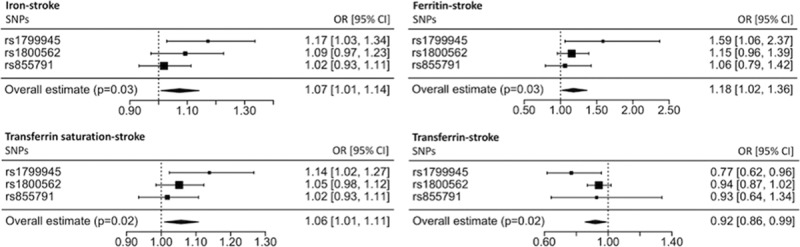
Main Mendelian randomization (MR) analysis estimates for each of the 4 biomarkers of iron status. The squares represent the individual single-nucleotide polymorphism (SNP) MR estimate, with their size proportional to the precision of the estimate and the 95% CIs represented by the horizontal lines. The diamonds represent the pooled SNP MR estimate, with the width indicating the 95% CIs. OR indicates odds ratio.

The results of the MR analysis, reported as odds ratios (ORs) of stroke per SD unit increase in each iron status biomarker, found a detrimental effect of an increased iron status on risk of any stroke for serum iron (OR, 1.07; 95% CI, 1.01–1.14; *P*=0.03), (log-transformed) ferritin (OR, 1.18; 95% CI, 1.02–1.36; *P*=0.03), and transferrin saturation (OR, 1.06; 95% CI, 1.01–1.11; *P*=0.02). In addition, a higher transferrin, indicative of a lower iron status, was associated with a decreased stroke risk (OR, 0.92; 95% CI, 0.86–0.99; *P*=0.02). The effect of each biomarker of iron status on risk of stroke is shown in Figure 1.

**Figure 2.**
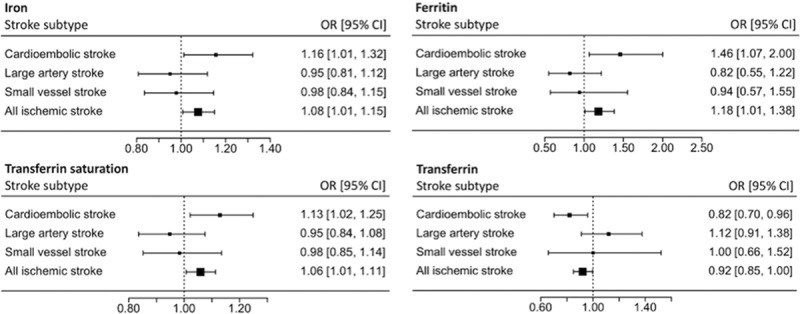
Main Mendelian randomization analysis estimates for each of the 4 biomarkers of iron status on risk of any ischemic stroke and ischemic stroke subtypes. OR indicates odds ratio.

Similar results were seen when investigating only ischemic stroke cases or European populations (Figures I and II, respectively, in the online-only Data Supplement). Investigating ischemic stroke subtypes revealed that the detrimental effect of iron status was driven by cardioembolic stroke (serum iron OR, 1.16; 95% CI, 1.01–1.32; *P*=0.03; [log-transformed] ferritin OR, 1.46; 95% CI, 1.07–2.00; *P*=0.02; transferrin saturation OR, 1.13; 95% CI, 1.02–1.25; *P*=0.02; transferrin OR, 0.82; 95% CI, 0.70–0.96; *P*=0.01). There was no significant effect of iron status on risk of large artery stroke (serum iron OR, 0.95; 95% CI, 0.81–1.12; *P*=0.54; [log-transformed] ferritin OR, 0.82; 95% CI, 0.55–1.22; *P*=0.32; transferrin saturation OR, 0.95; 95% CI, 0.84–1.08; *P*=0.41; transferrin OR, 1.12; 95% CI, 0.91–1.38; *P*=0.28). Furthermore, for the 2 SNPs available for small vessel stroke, neither was there any effect of iron status observed (serum iron OR, 0.98; 95% CI, 0.84–1.15; *P*=0.79; [log-transformed] ferritin OR, 0.94; 95% CI, 0.57–1.55; *P*=0.81; transferrin saturation OR, 0.98; 95% CI, 0.85–1.14; *P*=0.82; transferrin OR, 1.00; 95% CI, 0.66–1.52; *P*=0.998). The MR estimates for different ischemic stroke subtypes are compared in Figure 2, with results for individual SNPs given in Figures III–V in the online-only Data Supplement.

In our investigation of possible bias resulting from biological pleiotropy of the instruments, we found that the 3 SNPs were expectedly associated with iron status and red blood cell traits.^19^ A potentially detrimental pleiotropic effect on stroke risk may be conferred by rs1799945 because of the association of its iron status increasing allele with increased blood pressure.^19,22,23^ In addition, a protective effect on stroke risk may be conferred by the iron status increasing allele of rs1800562 because of its association with reduced total cholesterol and LDL (low-density lipoprotein) cholesterol levels.^19,22,24^ Investigating the any stroke and cardioembolic stroke outcomes using the 6 instrument SNPs selected by their genome-wide significant association with at least 1 iron status biomarker and associations with the remaining biomarkers in a pattern concordant with an effect on overall iron status (even if not reaching statistical significance) produced similar IVW MR estimates (Table). Consistency was also maintained when employing the MR-Egger and weighted median sensitivity analyses, and all analyses had concordant directions of effect with overlapping 95% CIs (Table). The MR-Egger approach did not produce evidence of directional pleiotropy in any of the analyses (for any stroke, iron *P*=0.85, ferritin *P*=0.59, transferrin saturation *P*=0.79, transferrin *P*=0.47; for cardioembolic stroke, iron *P*=0.12, ferritin *P*=0.51, transferrin saturation *P*=0.10, transferrin *P*=0.29).

## Discussion

### Findings in Context

We found evidence that an increase in genetically determined systemic iron status is associated with a higher risk of stroke and furthermore that this effect is driven by an increased risk of cardioembolic stroke. Numerous observational studies have previously reported a deleterious effect of higher iron status on risk of stroke.^5–7^ In contrast, several other studies have documented a link between iron deficiency and increased stroke incidence.^3–5,25–29^ The concept of both a reduced and increased iron status being associated with an increased stroke risk has been supported by the results of the National Health And Nutrition Examination Survey cohort.^5^ This study followed subjects for 12 years and included 652 cases of stroke, finding a significantly increased risk of stroke in white women at a transferrin saturation <30% and transferrin saturation >44% relative to those with a transferrin saturation of 30% to 36%.^5^ Although these results were not replicated in men,^5^ a Finnish cohort study with a 10-year follow-up period reported a lower risk of stroke in subjects with a serum iron concentration in the middle tertile compared with serum iron in the lowest tertile.^4^ Taken together, these studies may suggest a nonlinear association between iron status and stroke risk.^4,5^

A summary of the findings of observational studies investigating iron status and stroke risk are presented in Table II in the online-only Data Supplement. Only 2 case-control studies investigated the effect of iron status and risk of ischemic stroke subtypes, which is particularly relevant in view of our MR finding that the detrimental effect of higher iron status is driven by the cardioembolic stroke subtype. Chang et al^3^ found an increased risk of prior diagnosis of iron deficiency in cases of thrombotic and embolic stroke compared with healthy controls. A further case-control study investigating ischemic stroke subtypes found no significant association between risk of atherothrombotic, small vessel and cardioembolic stroke, and iron status biomarkers, including ferritin and transferrin saturation.^8^ The diverse population demographics and possible confounding factors may contribute to the heterogeneity of the results, along with the possibility of a nonlinear effect of iron status on stroke risk.

In terms of mechanistic insight, the contrast in our findings to those previously observed in an MR study of iron status and coronary artery disease suggests that the effect by which higher systemic iron status might increase risk of stroke is unlikely to be through atherosclerosis.^30^ Consistent with this, the analysis of ischemic stroke subtypes suggested that the detrimental effect of iron status is driven by an increased risk of cardioembolic stroke (Figure 2). Further work may, therefore, use the MR technique to investigate whether iron status also has a causal effect on risk of venous thromboembolism, which overlaps with cardioembolic stroke in the mechanism of thrombus formation, or indeed atrial fibrillation, which is the main risk factor for cardioembolic stroke. Indeed, GWAS meta-analyses for both of these phenotypes are available.^31,32^

Although higher iron status may not increase cardioembolic stroke risk through effects on atherosclerosis, iron-catalyzed reactions have been suggested to result in blood coagulation^33^ with the presence of dense fibrin-like deposits observed in the blood of patients with diabetes mellitus attributed to the prothrombotic action of free iron.^33^ To this end, it has been postulated that an excess of free iron in the blood results in the nonenzymatic generation of fibrin-like material that results in thrombus formation.^34^

### Strengths and Limitations

This study overcomes many of the limitations introduced in observational studies, by removing the impact of environmental confounding on the exposure and outcome.^11^ Indeed, ferritin levels rise, while transferrin levels fall in inflammation,^35^ and observational studies measuring these biomarkers may be confounded by concurrent inflammation. Furthermore, we were able to investigate differential effects of iron status in ischemic stroke subtypes. Indeed, this subtype analysis highlighted that the detrimental effect of iron status was driven by cardioembolic stroke. Of further interest was that for large artery stroke, a phenotype that has considerable pathophysiological overlap with coronary artery disease,^31^ rather than a detrimental effect of iron status, there may even be a possible suggestion of a protective effect (Figure 2). This is in keeping with the previous MR findings of a protective effect of iron status in coronary artery disease.^24^

A key assumption of MR is that there is a linear association between the change in the iron status biomarker and the risk of stroke^11^; however, this may not hold in the current context. For this reason, our MR results should not be extrapolated to extremes of iron status. Furthermore, by using genetic variants as markers of iron status, MR considers the lifetime effect of iron status.^11^ Hence, the predicted association between exposure and outcome may be greater in MR analysis compared with clinical practice.^11^ MR studies also carry the risk of pleiotropy.^11^ We investigated this by searching an online database for secondary effects of the main SNPs under investigation.^19^ As well as affecting iron status, some additional phenotypic effects of the SNPs were identified which may influence stroke risk independently of their effect on iron status. The effect of the iron status increasing allele of rs1799945 to increase systolic and diastolic blood pressure^19,23^ would be expected to exaggerate the detrimental effect of higher iron status because increased blood pressure has a deleterious effect on stroke risk.^22^ This is consistent with our results for all stroke, where rs1799945 generally had a larger effect on increasing risk of stroke compared with the overall IVW MR estimate (Figure 1). Furthermore, a protective effect of the iron status increasing allele of rs1800562 on stroke risk independent of any effect of this SNP on iron status may be related to its association with reduced total cholesterol and LDL cholesterol,^19,24^ which have both been associated with increased risk of ischemic stroke.^22^ Despite these possible sources of pleiotropy, all 3 SNPs had consistent associations with stroke risk, with overlap in the 95% CIs for the MR estimates for each SNP. This was most evident for cardioembolic stroke (Figure III in the online-only Data Supplement), where the individual MR estimates for each individual SNP were concordant in suggesting a detrimental effect of higher iron status.

We further tested the robustness of our findings to potential pleiotropy by relaxing our instrument selection criteria to increase the number of SNPs available for analysis so that statistical sensitivity tests could be performed. Specifically, SNPs for this were selected by their genome-wide significant association with at least 1 iron status biomarker and their association with the other biomarkers in a pattern consistent with an effect on overall iron status (ie, higher serum iron, ferritin, transferrin saturation, and lower transferrin), even if not at genome-wide significance. Although these more relaxed criteria may be more vulnerable to including SNPs that are not instruments of systemic iron status (but rather reflect levels of a single iron status biomarker), they increased the number of variants from 3 to 6. Consequent MR-Egger and weighted median analyses that are more robust to inclusion of pleiotropic variants also supported the main analysis albeit with their larger CIs attributable to lower statistical power (Table), thus adding further support to our results. Given the possible pleiotropic effect of the rs1799945 SNP used in the main IVW MR analysis through effects on blood pressure, these sensitivity analyses were crucial for demonstrating that our results were consistent in techniques that are more robust to the inclusion of pleiotropic variants. This is particularly important as approaches such as regression-based multivariable MR using summary data to adjust for the genetic effect of the instrument SNPs on blood pressure were not appropriate given that GWAS meta-analyses of blood pressure typically adjust for body mass index,^36^ which may itself bias these MR estimates.^37^ Furthermore, multivariable MR in this context may be limited by measurement error resulting in unreliable adjusted estimates,^38^ particularly as the number of instrument SNPs in this study is relatively small.

### Conclusions

This study investigated the association between iron status and stroke risk using MR and found that a higher iron status was associated with an increased risk of cardioembolic stroke. Further research should investigate the possible mechanism of effect and how this may be targeted towards preventative strategies.

## Acknowledgements

We thank the Genetics of Iron Status Consortium and the MEGSATROKE project for making the data used in this study publicly available. The MEGASTROKE project was funded by sources detailed at http://www.megastroke.org/acknowledgements.html, and the full list of authors is available in the Methods in the online-only Data Supplement.^16^ Dr Gill designed the study. Dr Gill and G. Monori performed statistical analysis. All authors interpreted the results. G. Monori and Dr Gill drafted the article. All authors critically revised the article for intellectual content. All authors approved the submitted version of the article. All authors are accountable for the accuracy and integrity of the work.

## Sources of Funding

Dr Gill is funded by the Wellcome 4i Clinical PhD Programme at Imperial College London.

## Disclosures

None.

## Supplementary Material
